# Crystallization-Dominated Rapid Setting of Geopolymers at Low-Preparation Temperature and Low-Modulus Alkali Activators

**DOI:** 10.3390/ma19040723

**Published:** 2026-02-13

**Authors:** Qingyun Liu, Tao Liu, Yimin Zhang, Qian Wan, Xiuqiong Fu

**Affiliations:** 1 School of Resource and Environmental Engineering, Wuhan University of Science and Technology, Wuhan 430081, China; lqy13687260549@163.com (Q.L.); zym126135@126.com (Y.Z.); wanqian@wust.edu.cn (Q.W.); xiuqiongfu@163.com (X.F.); 2 State Environmental Protection Key Laboratory of Mineral Metallurgical Resources Utilization and Pollution Control, Wuhan University of Science and Technology, Wuhan 430081, China; 3 Collaborative Innovation Center of Strategic Vanadium Resources Utilization, Wuhan University of Science and Technology, Wuhan 430081, China; 4 Hubei Provincial Engineering Technology Research Center of High Efficient Cleaning Utilization for Shale Vanadium Resource, Wuhan University of Science and Technology, Wuhan 430081, China

**Keywords:** geopolymers, rapid setting, crystallization, solid waste utilization

## Abstract

**Highlights:**

**What are the main findings?**

- A novel rapid-setting geopolymer preparation method is proposed.
- Setting time is reduced by over 90%.
- An activator crystallization-dominated setting mechanism is revealed.

**What are the implications of the main findings?**

- Provides an energy-saving solution for emergency engineering in cold climates.
- Opens a new avenue for rapid resource utilization of bulk solid waste.
- Establishes a new paradigm for designing setting-controllable geopolymers.

**Abstract:**

Current research on rapid-setting geopolymers primarily focuses on accelerating the “dissolution-gelation” reaction process (including dissolution, polycondensation, and hardening) to achieve the faster solidification of the material. In this work, the low-temperature crystallization phenomenon of sodium silicate solution is innovatively introduced into the geopolymer system, and a rapid-setting strategy is proposed by exploiting the synergistic effects of a low preparation temperature and a low activator modulus (the SiO_2_/Na_2_O molar ratio) to intentionally induce the rapid crystallization of the alkali activator solution (sodium silicate solution), thereby enabling rapid setting of the geopolymer. Experimental results show that, at a preparation temperature of 0–10 °C and an activator modulus of 1.0, the final setting time of the metakaolin-based geopolymer is shortened to 20 min, corresponding to a reduction of up to 91.63%. Unlike the typical “dissolution stage–gelation stage–hardening stage” route of conventional geopolymers, the rapid-setting geopolymer in this study follows a distinct reaction sequence of “hardening stage–dissolution stage–gelation stage”. Meanwhile, to further enhance performance and expand pathways for solid waste valorization, vanadium-extraction shale tailings (VST) were employed to partially replace metakaolin. The results indicate that, with a tailings replacement of 30%, an alkali-to-solid ratio of 0.4, and an alkali-to-water ratio of 0.7, the rapid-setting geopolymer achieves a compressive strength of 38.32 MPa. These findings confirm the broad applicability and practical potential of the proposed approach for emergency repair in cold regions and solid waste utilization.

## 1. Introduction

The demand for rapid-setting materials continues to grow across various engineering fields [1]. In emergency repairs of critical infrastructure like airport runways and bridge decks [2,3], rapid-setting materials can significantly reduce traffic closure times. For underground engineering and shotcrete applications [4], controlled rapid setting ensures timely ground support and enhances construction safety. Furthermore, in emerging technologies like 3D concrete printing [5,6], the precise control of setting time is essential for maintaining the pumpability of the paste and achieving rapid structural build-up after extrusion. While ordinary Portland cement (OPC) systems typically employ various admixtures to achieve these properties, geopolymers demonstrate significant potential as a sustainable alternative to OPC in these demanding advanced applications [7,8,9], offering superior durability and a lower carbon footprint [10,11,12,13]. Therefore, extending the application of geopolymers to the field of rapid-setting materials holds important practical significance.

Current mainstream methods for accelerating the setting of geopolymers primarily focus on speeding up the dissolution, polymerization, and reprecipitation processes of the geopolymerization reaction (dissolution-gelation). Research has mainly concentrated on aspects such as raw material composition, alkaline activator characteristics, and curing regimes [14,15]. Regarding raw materials, it has been reported that incorporating high-calcium slag or furnace slag can effectively shorten the setting time [16,17]. Ma et al. indicated that [18] increasing the blast-furnace slag content from 20% to 50% reduced the initial setting time by approximately 150 min, which was attributed to Ca^2+^ promoting the rapid formation of cementitious phases. With respect to alkali activators, both the activator type and the silicate modulus significantly affect the setting behavior of geopolymers [14,19]. Potassium-based geopolymers generally harden more slowly than sodium-based systems because smaller cations tend to interact more readily with silicate oligomers [20,21]. Reducing the silicate modulus of the activator mainly accelerates setting by increasing the alkalinity of the system, thereby enhancing the dissolution of aluminosilicate precursors [19]. In terms of curing regimes, elevating the curing temperature is among the most direct and effective means to shorten setting time [22]. Mo et al. reported that increasing the curing temperature from 20 °C to 100 °C reduced the final setting time of geopolymer paste from 22 h to 20 min [23]. This is achieved by supplying additional activation energy, which markedly accelerates precursor dissolution and the subsequent gelation process.

In inorganic salt systems, the low-temperature crystallization of solutes is of particular interest and offers a new perspective for rapid solidification of geopolymers. Crystallization [24,25] is a ubiquitous physicochemical process, the essence of which lies in creating supersaturation to induce solute precipitation and the formation of an ordered solid structure; this process can effectively immobilize free water and provide immediate rigidity. Sodium silicate, a commonly used geopolymer raw material, has been reported to exhibit low-temperature crystallization behavior [26]. In separation science and engineering, quench crystallization is often employed to precipitate hydrated sodium metasilicate crystals (Na_2_SiO_3_·nH_2_O) from concentrated sodium silicate solutions for the recovery of silicon and sodium resources. When investigating the temperature effect on geopolymers, existing studies typically focus on curing temperature, while largely overlooking the preparation temperature (i.e., the ambient temperature experienced at all stages prior to curing, including the storage temperature of raw materials and alkali activators and the temperature during their mixing). In geopolymer production, highly concentrated sodium silicate solutions are frequently used as alkali activators; their crystallization behavior under low-temperature conditions provides an innovative route toward rapid geopolymer hardening.

Based on this fundamental principle, this study proposes an innovative method for preparing rapid-setting geopolymers through physical crystallization. It investigates the effects of preparation temperature and alkali activator modulus on the setting time and compressive strength of geopolymers, revealing the synergistic mechanism of low temperature and low modulus in inducing sodium silicate crystallization to achieve rapid setting. Furthermore, vanadium-bearing shale tailings [27] (VST) are inherently rich in silicate and aluminate mineral components required for geopolymer preparation. Through appropriate activation treatment, VST can be transformed into high-performance cementitious materials. This approach not only enables the reduction and high-value utilization of tailings, alleviating the environmental pressure associated with stockpiling, but also yields geopolymer materials with excellent mechanical properties that meet the strength requirements for construction applications. Therefore, this manuscript further explores the influence of VST as a raw material on the performance of rapid-setting geopolymers. The study aims to provide novel research insights for achieving rapid setting in geopolymers and promoting the resource utilization of solid waste.

## 2. Materials and Experimental Methodology

### 2.1. Materials

**Figure 1:**
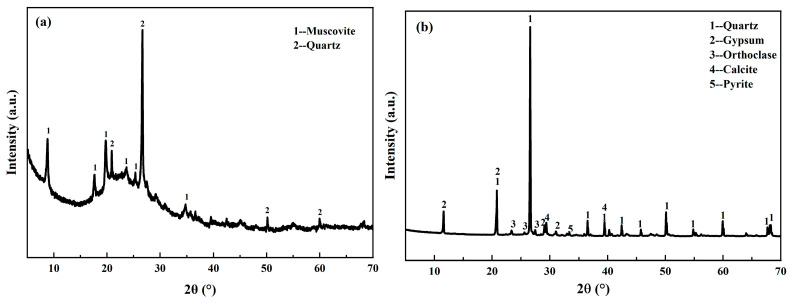
XRD pattern of MK (**a**) and VST (**b**).

Kaolin was calcined at 800 °C [28,29] for 6 h to obtain higher reactivity, producing metakaolin (MK) as the raw material for the geopolymer reaction. The chemical composition and XRD results of MK are shown in Table 1 and Figure 1, respectively. The results indicate that the primary chemical components of MK are SiO_2_ and Al_2_O_3_. Apart from an amorphous hump, the main crystalline phases identified were muscovite and quartz. The vanadium-bearing shale tailings (VST) used in this study were obtained from a mining company in Shaanxi Province, China. Its primary components are quartz and gypsum, with detailed chemical composition and phase composition presented in Table 1 and Figure 1, respectively. The alkali activator solution employed was prepared by mixing powdered instant sodium silicate (modulus 3) with sodium hydroxide and water. Sodium silicate and sodium hydroxide were purchased from Shandong Keyuan Biochemical Co., Ltd (Heze, China). and Sinopharm Chemical Reagent Co., Ltd. (Shanghai, China), respectively. Deionized water was used throughout all experimental procedures.

### 2.2. Synthesis of Geopolymer

This experiment was conducted at two preparation temperatures (the ambient temperature at all stages prior to curing, including the storage temperature of raw materials and alkali activators, as well as the temperature during their mixing). At both preparation temperatures, the modulus of the alkali activator was adjusted to 0.8, 1.0, 1.2, 1.5, and 2.0 by varying the ratio of sodium silicate to sodium hydroxide, while maintaining a constant molar quantity of Na_2_O at 1 mol in the solution. The raw materials and alkali activators of different moduli were mixed for 90 s using a mechanical stirrer (ARE-310) at 2000 rpm to obtain fresh geopolymer slurry. The slurry was poured into PTFE molds (30 × 30 × 30 mm^3^) and vibrated for two min to release air bubbles. All geopolymers were cured at 25 °C. Geopolymers prepared at low temperature using activators with moduli of 0.8, 1.0, 1.2, 1.5, and 2.0 were denoted as L-GM0.8, L-GM1.0, L-GM1.2, L-GM1.5, and L-GM2.0, respectively. Those prepared at normal temperature using activators with moduli of 0.8 and 1.0 were denoted as N-GM0.8 and N-GM1.0, respectively. The detailed mix proportions and preparation temperatures for metakaolin-based geopolymers are summarized in Table 2.

### 2.3. Characterization

The chemical composition of metakaolin (MK) was determined by inductively coupled plasma atomic emission spectroscopy (ICP-AES, PerkinElmer, Massachusetts, USA). The setting times of geopolymers were measured using a TKS-1 Vicat apparatus (Shangyu Prospecting Company, Shaoxing, China). The compressive strength of the geopolymers was evaluated using a YAW-300 mechanical tester (Jinan Hensgrand Instrument Company, Jinan, China). At least three samples were tested, and the average values were used. The crystal structure of the geopolymers was characterized by X-ray diffraction (XRD, Rigaku D/MAX 2500PC, Takatsuki, Japan) using Cu Kα radiation. The morphology of the geopolymers was examined by scanning electron microscopy equipped with an energy dispersive spectrometer (SEM-EDS, JEOL JSM-IT300, Tokyo, Japan). The chemical bonding information of MK and the geopolymers was analyzed using a Fourier-transform infrared spectrometer (FTIR, Nexus Thermo Nicolet, Madison, WI, USA). Transmittance spectra were collected over the wavenumber range of 400–4000 cm^−1^ with a resolution of 2 cm^−1^. Low-field NMR T_2_ measurements were performed using the Carr-Purcell-Meiboom-Gill (CPMG) pulse sequence with the following parameters: echo time of 0.1 ms, 2000 echoes, a repetition interval of 3000 ms, and 8 accumulated scans. A specific surface area and pore size analyzer (JW-BK100C, Beijing, China) was employed to perform nitrogen adsorption/desorption measurements on the soil polymer samples based on the Barrett-Joyner-Halenda (BJH) method. This yielded desorption isotherms and pore volume distributions within the 1–100 nm range. The form of water in the samples was tested using a simultaneous thermal analyzer (TGA-DSC, Mettler Toledo, Zurich, Switzerland). The temperature of the samples was gradually increased from 50 °C to 500 °C in a corundum crucible under nitrogen, a high-purity dynamic inert gas, at an average rate of 10 °C/min.

## 3. Results and Discussion

### 3.1. Regulation of Rapid-Setting Conditions in Metakaolin-Based Geopolymers

**Figure 2:**
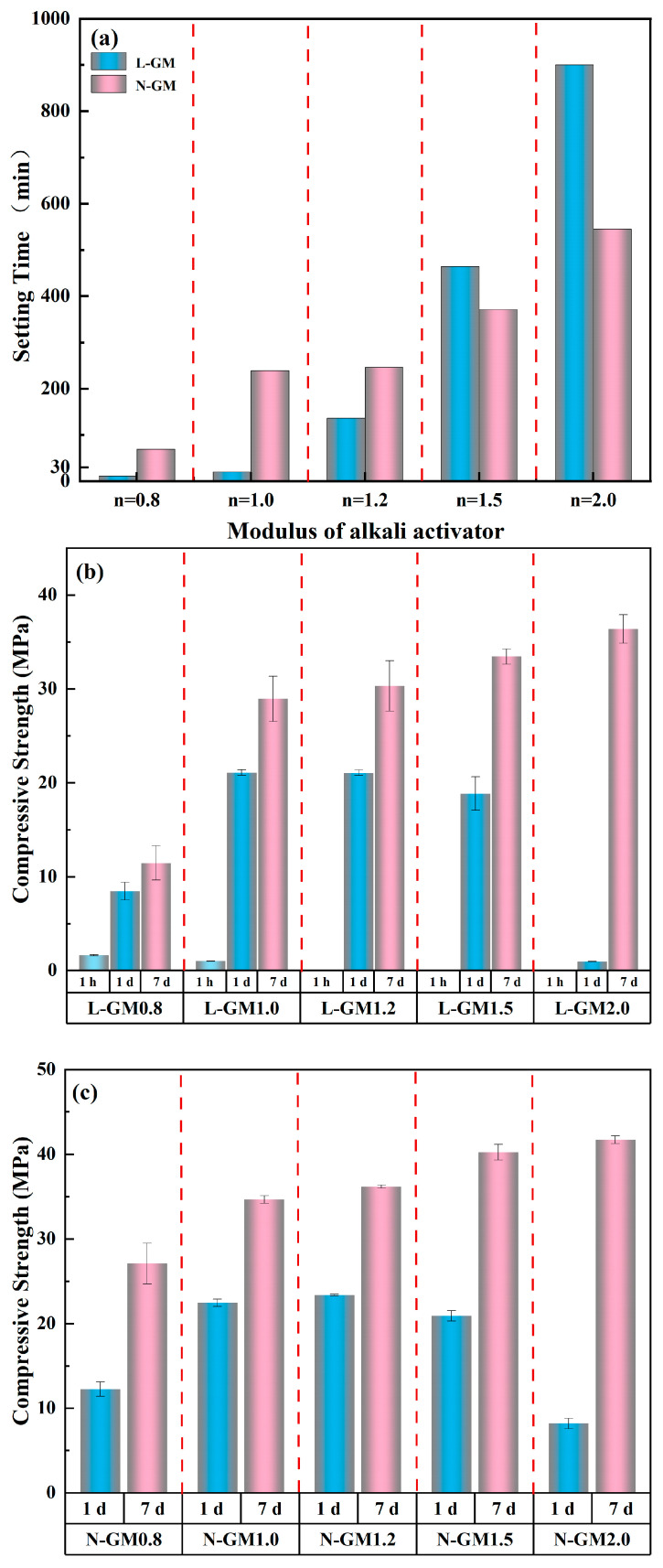
(**a**) Setting time under different moduli at various preparation temperatures. (**b**) Compressive strength under different moduli at low preparation temperature. (**c**) Compressive strength under different moduli at normal preparation temperature.

Figure 2a shows the variation in setting time with activator modulus for geopolymers prepared at low temperature (L-GM) and at ambient temperature (N-GM). As can be seen, the setting time generally increases with increasing activator modulus. This is because a higher modulus indicates a higher content of soluble silica in the solution while the relative content of effective alkali decreases, thereby slowing the dissolution of metakaolin and prolonging the setting time [19]. In general, lower temperature hinders the dissolution, polymerization, and reprecipitation processes involved in the reaction, resulting in an extended setting time. When the activator modulus is greater than 1.2, the setting behavior follows this rule, i.e., N-GM exhibits a shorter setting time than L-GM. However, when the activator modulus is ≤1.0, the setting time of L-GM becomes markedly shorter than that of N-GM. Specifically, at a modulus of 0.8, the setting time of L-GM0.8 is only 11 min, far shorter than the 69 min of N-GM0.8, corresponding to an 84.06% reduction. At a modulus of 1.0, the setting time of L-GM1.0 is only 20 min, again much shorter than the 239 min of N-GM1.0, representing a 91.63% reduction. These results indicate that the rapid-setting phenomenon (rapid hardening of geopolymer paste within 30 min) is related to both activator modulus and preparation temperature, and is likely attributable to their synergistic effect.

Figure 2b,c present the compressive strength of geopolymers with different activator moduli prepared at different temperatures. As shown, the compressive strength of L-GM is consistently lower than that of N-GM, which is associated with the inhibitory effect of low temperature on geopolymerization to some extent. For samples with an activator modulus of 0.8, the 7 d compressive strength of L-GM0.8 is only 11.48 MPa, compared with 27.10 MPa for N-GM0.8, representing a decrease of 57.64%. This suggests that, although low preparation temperature can effectively reduce the setting time and achieve rapid setting for geopolymers with a modulus of 0.8, it leads to a pronounced loss in compressive strength. For samples with an activator modulus of 1.0, the 7 d compressive strength of L-GM1.0 is 28.95 MPa, which is only 5.72 MPa lower than that of N-GM1.0 (34.67 MPa). This indicates that, for geopolymers with a modulus of 1.0, low preparation temperature can substantially shorten the setting time and enable rapid hardening while maintaining a considerable compressive strength. The above results demonstrate that the crystallization-dominated mechanism offers significant advantages in achieving ultrafast setting. By modulating the modulus under low preparation temperature, a balance between rapid setting and relatively high strength can be attained.

### 3.2. Phase and Chemical Bond Characterization

**Figure 3:**
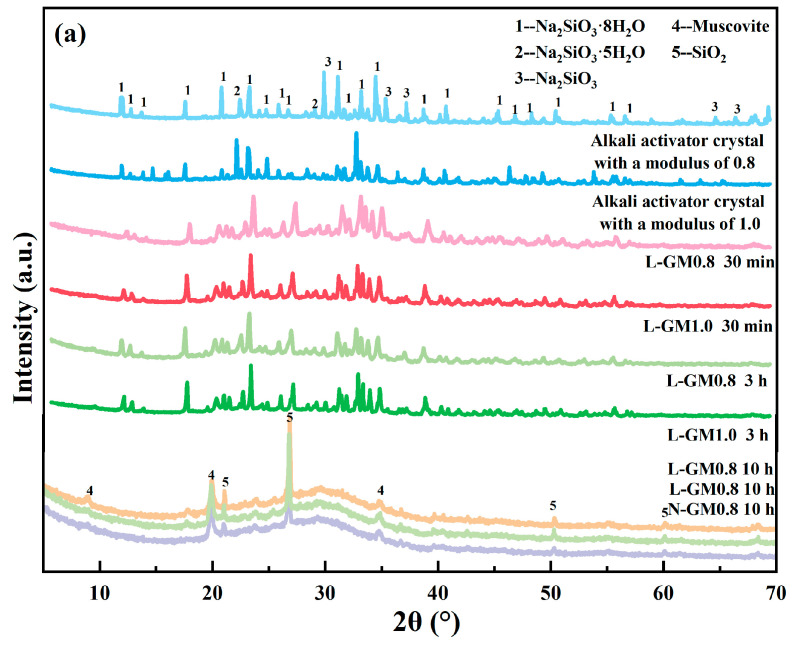

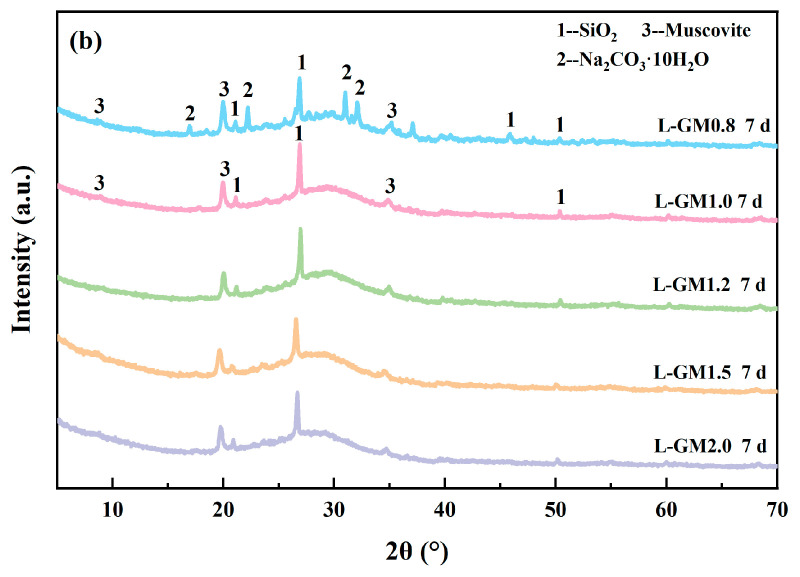
XRD patterns of (**a**) Alkali activator crystal, L-GM0.8 and L-GM1.0, (**b**) L-GM.

To investigate the reasons behind the rapid setting of geopolymers under low temperature and low modulus conditions, it is necessary to study their phase composition, chemical structure, and other relevant aspects. Figure 3a presents the XRD patterns of crystals precipitated from the alkali activator solutions and of L-GM0.8 and L-GM1.0 cured for different durations. The primary phase of the precipitated crystals is sodium silicate hydrate. Compared to L-GM1.0, L-GM0.8 exhibits more numerous and diverse crystal peaks, which may account for the faster setting of L-GM0.8. After 30 min of curing, crystalline peaks of sodium silicate hydrate are observed in both L-GM0.8 and L-GM1.0. Notably, the characteristic amorphous hump of geopolymers in the range of 25–30° had not yet formed at this stage. This indicates that the rapid setting of L-GM0.8 and L-GM1.0 is not primarily driven by the geopolymerization reaction but is instead caused by the crystallization of sodium silicate hydrate [30]. This may occur because low-modulus alkali activators form sodium silicate hydrate crystals when mixed with raw materials at low temperatures, converting abundant free water into crystalline water and thereby eliminating slurry fluidity. In contrast, high-modulus alkali activators predominantly contain silicate polymers that are less prone to sodium silicate crystallization [31,32]. After 10 h of curing, amorphous peaks characteristic of geopolymers are formed in both L-GM0.8 and L-GM1.0, which is also observed in N-GM1.0 after the same curing period, indicating a similar phase composition. This suggests that geopolymerization can proceed even after crystallization of the alkali activator. Figure 3b shows the XRD patterns of geopolymers with different moduli cured for 7 days at low preparation temperatures. The results show that all L-GM samples exhibited similar amorphous humps characteristic of geopolymers after 7 days of curing, with crystalline peaks primarily attributed to quartz and mica. In the diffractogram of the L-GM0.8 sample, distinct crystalline peaks corresponding to Na_2_CO_3_·10H_2_O are observed, which are attributable to carbonation of the geopolymer. This may result from the excessively high alkalinity of L-GM0.8, where excess alkali reacts with atmospheric CO_2_ in the presence of residual water in the specimen [33,34]. This carbonation-related product formation is also considered a key reason why the compressive strength of L-GM0.8 is lower than that of L-GM1.0.

**Figure 4:**
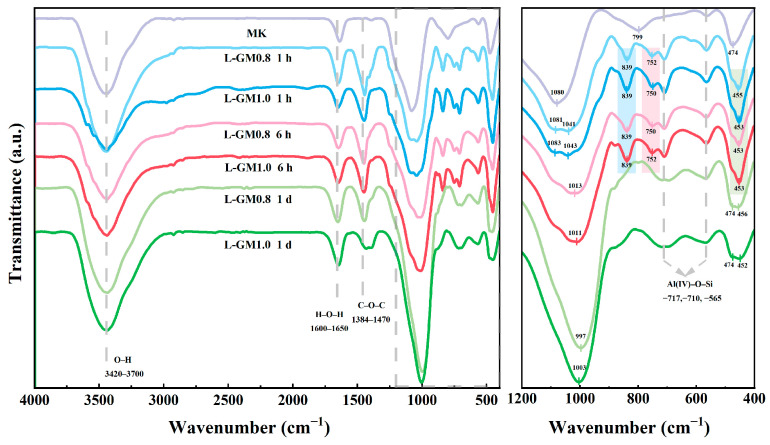
FTIR spectra of LGM0.8 and LGM1.0.

To elucidate the influence of hydrated crystallization on the geopolymerization process, FTIR spectroscopy was employed to examine the evolution of chemical bonding in L-GM samples at different curing ages. Figure 4 gives the FTIR spectra of L-GM0.8 and L-GM1.0 at different curing ages. The absorption peaks near 3420–3700 cm^−1^ and 1600–1650 cm^−1^ are attributed to OH^−^ stretching vibrations and H-OH bond vibrations from free water in MK or alkali activators [35]. The peak observed around 1384–1470 cm^−1^ is caused by carbonate vibration, resulting from carbonation during geopolymer synthesis [36]. The peaks at 799 cm^−1^ and 474 cm^−1^ represent the stretching vibration of 4-coordinated Al(IV)-O and the O-Si-O vibration in MK. The disappearance of these peaks is associated with the depolymerization of MK [37,38]. The peak at 1080 cm^−1^ and 717 cm^−1^ in the FTIR spectrum of MK is due to the asymmetric and symmetric stretching modes of Si-O-Si and Si-O-Al bonds [37]. The persistence of this peak in the spectrum after 1 h of curing indicates incomplete dissolution of MK at this stage. The band at 840 cm^−1^ can be attributed to the typical Si-O stretching vibration in Q^1^ chain structures, which is likely due to the crystallization of sodium silicate [39]. The peak at 752 cm^−1^ originates from the parallel vibration of Al-OH, potentially related to tetrahedral monomers of aluminum dissolved from the raw material [40]. These peaks disappear after 1 day of curing, indicating that the reaction between sodium silicate and metakaolin was essentially complete by this time, which is consistent with the XRD results.

### 3.3. Microstructural Characteristics and Pore Structure

**Figure 5:**
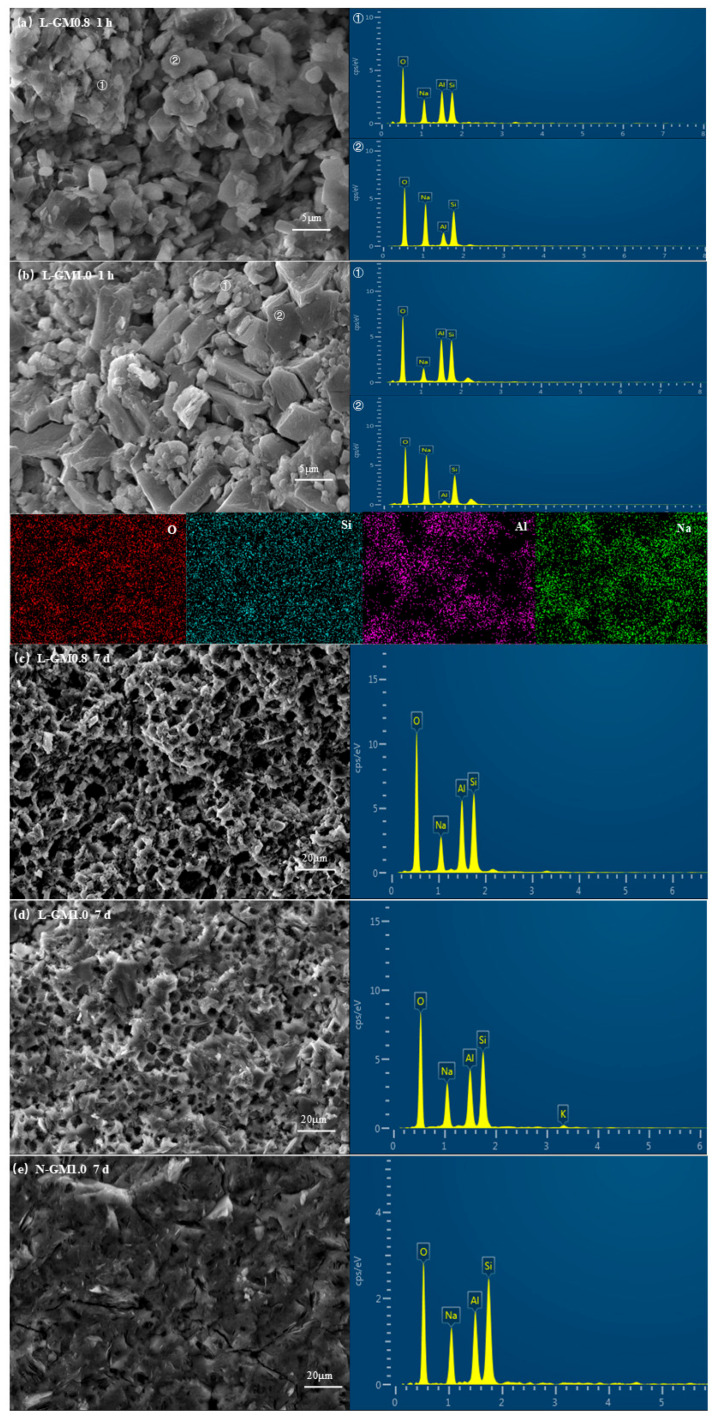
The SEM images and EDS analysis of L-GM0.8-1 h (**a**), L-GM1.0-1 h (**b**), L-GM0.8-7 d (**c**), L-GM1.0-7 d (**d**), N-GM1.0-7 d (**e**).

Based on the phase assemblage identified by XRD, the microstructural morphology and elemental distribution of the rapid-setting geopolymers were further characterized by SEM-EDS, as shown in Figure 5. As shown in Figure 5b, distinct monoclinic crystalline structures can be observed in L-GM1.0 after 1 h of curing, with unreacted raw particles embedded within the intercrystalline voids. EDS analysis reveals sodium predominantly distributed within the crystalline phase, exhibiting a distribution pattern largely opposite to that of aluminum. This indicates that geopolymer gel formation has not yet occurred at this stage, and the early strength of the rapid-setting geopolymer is derived from the physical framework formed by hydrated sodium silicate crystals. Meanwhile, at the same magnification, the crystals observed in the L-GM0.8 specimen are smaller in size and more abundant than those in the L-GM1.0 specimen, and they are predominantly stacked. This is likely because the activator with a modulus of 0.8 possesses higher silicate supersaturation, leading to a faster crystallization rate during the formation of hydrated sodium silicate crystals. This explains the shorter setting time of the L-GM0.8. After 7 days of curing, The SEM images of L-GM0.8 clearly reveal pores of varying sizes, resulting in an excessively porous structure of the geopolymer gel. In contrast, the number of pores in L-GM1.0 is significantly reduced, allowing most of the gel to cross-link-explaining its superior compressive strength. However, the N-GM1.0 sample exhibits virtually no large pores, presenting a uniformly dense gel. Therefore, it is inferred that the pores in the rapid-setting geopolymers (L-GM0.8 and L-GM1.0) may originate from voids left after crystal dissolution that are not completely filled by the gel network. At a lower modulus, the increased crystal formation results in higher porosity and consequently lower strength.

**Figure 6:**
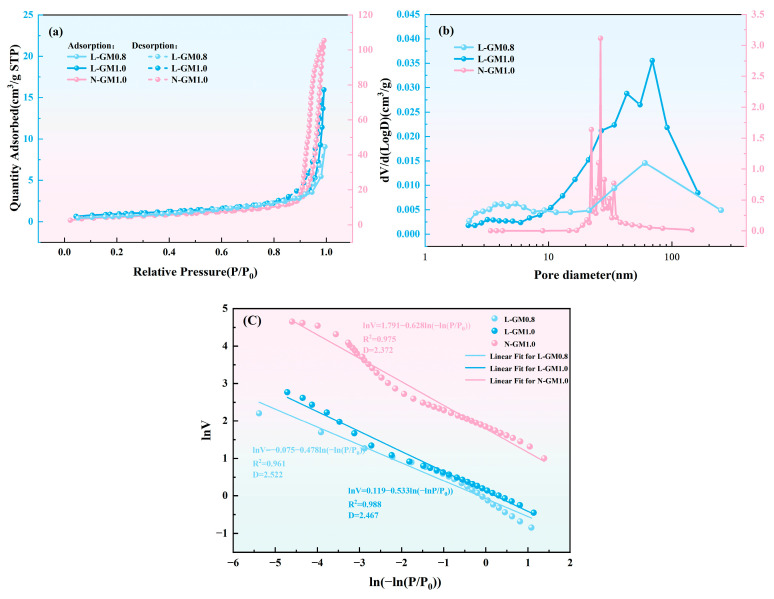
(**a**) N_2_ adsorption and desorption isotherm and (**b**) Pore characteristics. (**c**) Fractal dimension of pore size distribution.

Figure 6a presents the N_2_ adsorption–desorption isotherms and pore size distributions of L-GM0.8, L-GM1.0, and N-GM1.0 after curing for 14 days. The results show that all samples exhibit a clear separation between the adsorption and desorption branches in the relative pressure (P/P_0_) range of 0.8–1.0, indicating the presence of pronounced hysteresis loops. This behavior corresponds well with the pore size distribution shown in Figure 6b [41]. Notably, no plateau is observed in the isotherms even at relative pressures approaching the saturated vapor pressure, suggesting that adsorption does not reach saturation and that the samples possess a continuous pore network. As shown in Figure 6b, N-GM1.0 exhibits a more concentrated pore size distribution, with significantly higher specific surface area and pore volume compared to L-GM0.8 and L-GM1.0; the detailed textural parameters are summarized in Table 3. The fractal dimension (D) is an important parameter for characterizing the irregularity of solid surfaces and pore structures; a higher fractal dimension indicates stronger heterogeneity and a broader pore size distribution [42]. Figure 6c shows the regression results obtained using the Frenkel–Halsey–Hill (FHH) model for fractal dimension calculation. The results show that L-GM0.8 exhibits the highest fractal dimension (2.52), whereas N-GM1.0 shows the lowest value (2.37), indicating that the pore structure of L-GM0.8 is more complex and heterogeneous, which may imply potential challenges in terms of durability. By contrast, L-GM1.0 achieves a more favorable balance between curing rate and macroscopic strength compared with L-GM0.8.

### 3.4. Analysis of Thermal Behavior and Reaction Mechanism

**Figure 7:**
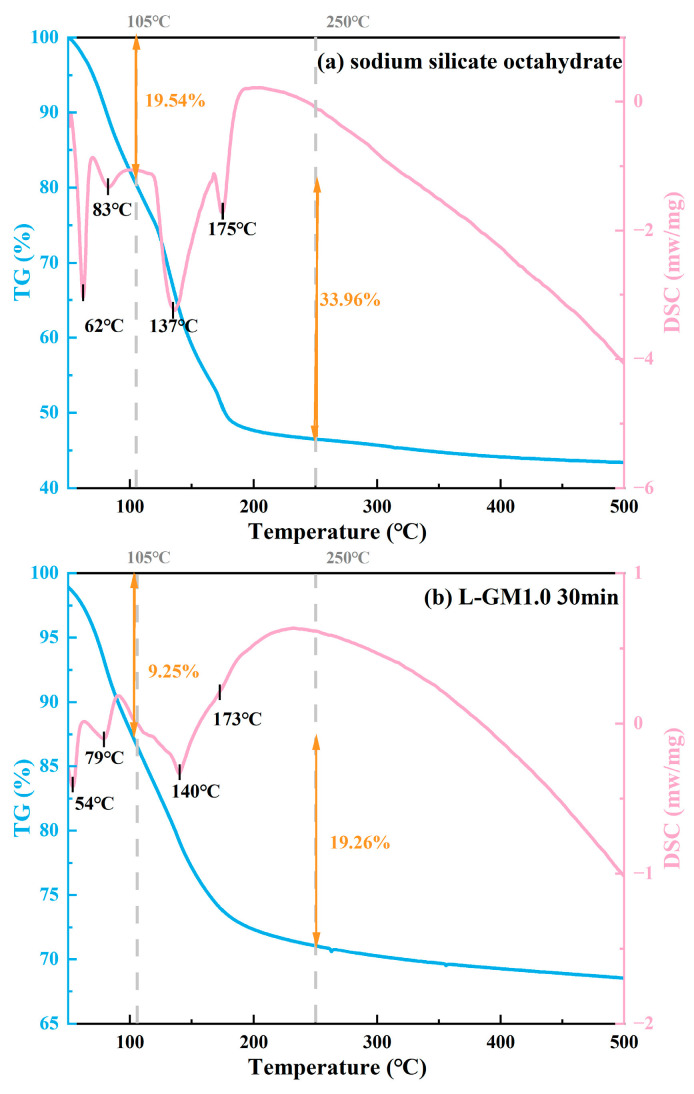

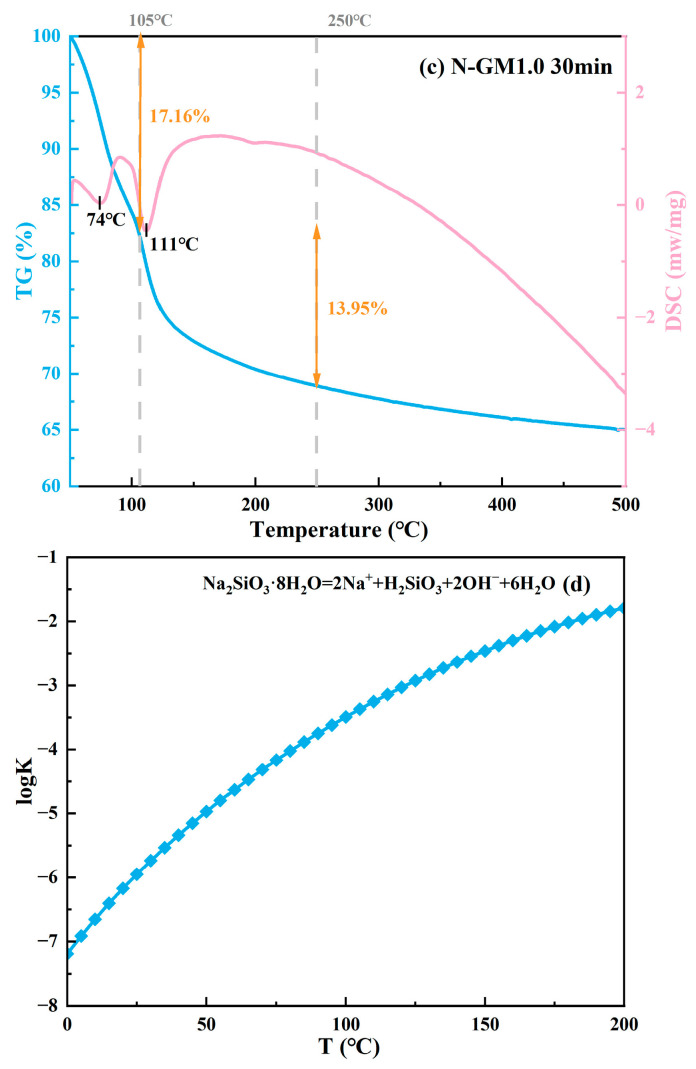
TG-DSC curves of sodium silicate octahydrate (**a**), L-GM1.0-30min (**b**), N-GM1.0 (**c**), logK-T curves (**d**).

Figure 7a shows the TG-DSC curves of crystals precipitated from the alkaline activator solution. The mass loss occurred predominantly within 50–250 °C and can be divided into two stages. The first stage, from 50 to 105 °C, exhibited a mass loss of approximately 19.54%, while the second stage, from 105 to 200 °C, showed a loss of about 33.96%. This value agrees well with the theoretical mass fraction of crystallization water in Na_2_SiO_3_·8H_2_O (about 54.14%), indicating that the precipitated phase is mainly sodium metasilicate octahydrate. Moreover, the mass losses in the first and second steps correspond to the loss of approximately 3 and 5 molar equivalents of crystallization water, respectively. The DSC curve of the L-GM1.0–30 min sample exhibited an endothermic peak similar to that of sodium metasilicate octahydrate, further confirming the early precipitation of crystalline phases in the low-temperature system. Its first-stage mass loss was only 9.25%, much lower than that of the N-GM1.0-30min sample (17.16%), suggesting that at least 7.91% of the L-GM1.0 sample participated in the reaction corresponding to the second-stage mass loss of hydrated sodium silicate crystallization. Based on the calculation from water loss associated with crystal water, at least 4.55% of the water lost in the first stage of L-GM1.0 originated from crystal dehydration. Figure 7d presents the temperature dependence of the equilibrium constant (logK) for the decomposition of Na_2_SiO_3_·8H_2_O, calculated using HSC Chemistry. As the temperature increases, the Log K value shows an increasing trend, indicating that elevated temperature favors the dissolution of sodium metasilicate octahydrate. This suggests that the sodium silicate crystals formed under low preparation temperature conditions can reach a new dissolution equilibrium during the curing stage at 25 °C. At this stage, the combined effects of the rising pore solution temperature and the reaction consumption (the dissolution of metakaolin in the alkaline environment and the polycondensation of silicate/aluminate monomers) can drive the continuous dissolution of the early-precipitated hydrated sodium silicate crystals. This process ultimately redirects the system’s reaction pathway back to that observed in the system prepared at normal temperature.

**Figure 8:**
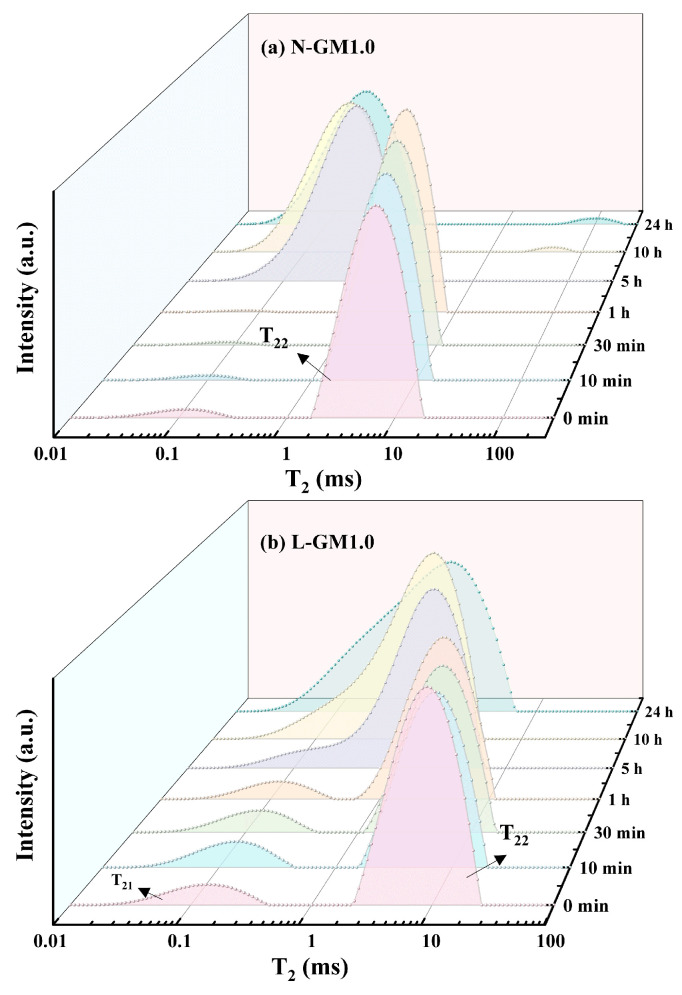
T_2_ distributions of N-GM1.0 (**a**) and L-GM1.0 (**b**).

To elucidate the curing process of rapid-setting geopolymers, the T_2_ distributions within the first day were measured for geopolymer samples with a modulus of 1.0 prepared at low temperatures (L-GM1.0) and at conventional temperatures (N-GM1.0), respectively. The results are presented in Figure 8, and the corresponding data are summarized in Table 4. The evolution of T_2_ relaxation and signal intensity is closely associated with the spatial location of water in the geopolymer matrix and its relative abundance; a smaller T_2_ indicates more strongly bound water or smaller pores [43,44]. In general, geopolymer pastes exhibit a unimodal T_2_ signal that progressively shifts to shorter times (leftward) as the reaction proceeds. At the beginning, water is mainly located in capillary pores (T_2_ > 1.0 ms). With ongoing reaction, water is predominantly redistributed into the nanopore space of reaction products (gel), referred to as nanopore water (T_2_ = 0.1–1 ms). When most of the water has transferred into the nanopore domain, the geopolymerization reaction is considered essentially complete.

The initial signal was collected after the mixing of raw materials with the alkali activator. For the N-GM1.0 sample, the reaction can be divided into three stages. In Figure 8a, a relatively low initial S_0_ at 0 min is observed, providing indirect evidence of a dissolution period prior to data collection [44]. During this period, metakaolin reacts with water and sodium silicate to generate several Si and Al monomeric species; consequently, part of the water is consumed as a reactant, leading to a decrease in S_0_. The second stage (0–5 h) corresponds to a dehydration process associated with gelation [45], which is reflected by an increase in S_0_. During gelation, Si and Al monomers progressively form a gel phase, as evidenced by the decrease in T_22_. The third stage, after 5 h, is the setting stage, characterized by a decrease in both T_22_ and S_0_. This is attributed to the binding of water with the gel [46] and the polycondensation reactions between gels [47].

For the L-GM1.0 sample, the reaction process differs. In Figure 8b, an additional T_21_ peak (0.01–0.33 ms) emerges, which is attributed to crystallization water in hydrated sodium silicate formed during mixing of the precursor and activator. The first stage, from the onset of mixing to 10 min, is a crystallization-burst period: the T_21_ intensity increases while the T_22_ intensity decreases, indicating continuous conversion of capillary water into crystal water. Macroscopically, the paste rapidly loses flowability and exhibits rapid setting. This stage is dominated by physical processes, and the early strength of the rapid-setting geopolymer primarily originates from a physical skeleton formed by intergrown crystals. The second stage, from 10 min to 1 h, is marked by a deceleration or cessation of crystallization, during which metastable crystals begin to decompose initially. This phase is evidenced by a decrease in T_21_ intensity, suggesting the commencement of release for crystal water. Concurrently, high-concentration hydroxide and silicate ions released from crystal dissolution erode raw material particles, liberating silicon and aluminum monomers. The T_22_ peak position continuously shifts leftward, migrating from 1.65 ms to 0.62 ms, indicating persistent pore space reduction as gel phases begin forming and occupying space. This stage represents a competitive and transitional period between physical and chemical processes, where crystals start decomposing to supply raw materials for geopolymerization. The third stage is identified as the gelation stage from hour 1 to hour 5, characterized by the merging of the two peaks and a significant increase in S_0_. During this period, the original T_21_ and T_22_ peaks merge into a new T_22_ peak, indicating blurred boundaries between the crystalline and gel phases. The gel progressively fills the physical framework constructed by crystals, transitioning the system toward a predominantly gel-dominated structure. However, due to the asynchronicity and inhomogeneity of crystal decomposition and raw material dissolution, coupled with the low fluidity of the system, the gel cannot freely expand and form a homogeneously cross-linked network as typically observed in conventional systems cured at ambient temperature. The final stage can be further divided into two periods separated at 10 h. From 5 to 10 h, the T_22_ peak shifts from 2.10 ms to 1.40 ms, indicating continued pore refinement; by 10 h, the pore structure becomes essentially stabilized, implying that the crystalline phase has largely decomposed, consistent with the XRD and FTIR results. After 10 h, the T_22_ peak position remains unchanged, whereas the continued increase in S_0_ confirms that polycondensation within the gel network proceeds slowly. At 24 h, the T_22_ peak of L-GM1.0 is noticeably broader than that of N-GM1.0, indicating a wider pore-size distribution and poorer uniformity, in agreement with the previously obtained fractal dimension (D) results.

### 3.5. Preparation of Rapid-Setting Geopolymers Using VST

The above findings demonstrate that rapid setting of metakaolin-based geopolymers can be successfully achieved through a crystallization-dominated solidification mechanism driven by sodium silicate, and that this effect can be effectively regulated by activator modulus and preparation temperature. T This study further investigates the influence of vanadium-bearing shale tailings (VST) as a raw material on the performance of rapid-setting geopolymers. Considering that a modulus of 1.0 provided an optimal balance between sufficient alkalinity and crystallization induction in the metakaolin system, the sodium silicate modulus was fixed at 1.0 for the tailings-based geopolymers, with the preparation temperature controlled within 0–10 °C.

**Figure 9:**
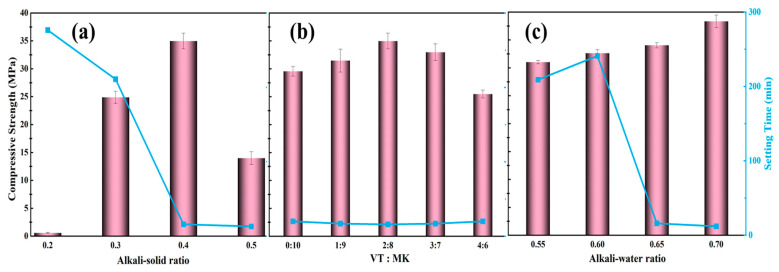
Single-factor experiments on the preparation of geopolymers from vanadium shale tailings: (**a**) A/S, (**b**) VST/MK, and (**c**) A/W.

**Figure 10:**
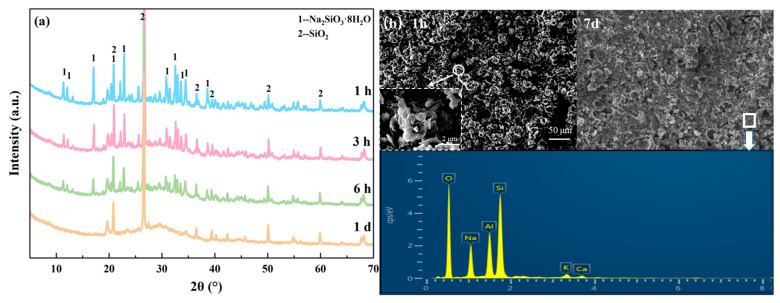
Rapid-setting geopolymer sample: (**a**) XRD spectrum, (**b**) SEM-EDS spectrum.

**Figure 11:**
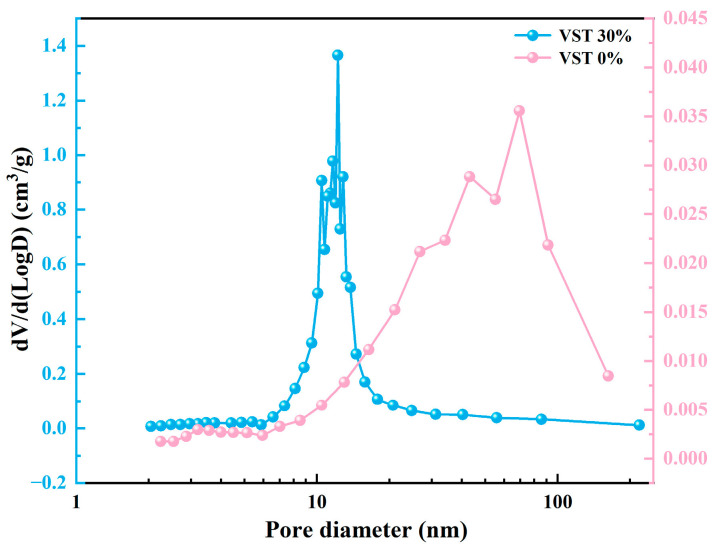
Pore characteristics.

The synthesis of rapid-setting geopolymers from shale vanadium extraction tailings was designed based on three key parameters: alkali-to-solid ratio, VT:MK ratio, and alkali-to-water ratio. The alkali-to-solid ratio refers to the ratio of the total mass of alkali activators (NaOH + Na_2_SiO_3_) to the total mass of solid precursors (MK + VST). The results are shown in Figure 9. As illustrated in Figure 9a, rapid setting occurred when the alkali-to-solid ratio was ≥0.4, indicating that adequate sodium silicate crystallization is required to rapidly immobilize the geopolymer slurry; however, excessive alkali dosage impairs strength development. Figure 9b shows that the incorporation of an appropriate amount of VST can effectively enhance the compressive strength of rapid-setting geopolymers, which is attributed to the adjustment of the Si/Al ratio in the system induced by VST addition. Figure 9c indicates that rapid setting was observed when the alkali-to-water ratio exceeded 0.65, as a lower alkali-to-water ratio results in excessive free water, limiting its conversion to crystalline hydration products. When the alkali-to-water ratio is 0.70, the compressive strength of the geopolymer can be increased to 38.32 MPa. A sample with an alkali-to-solid ratio of 0.4, a VT:MK ratio of 3:7, and an alkali-to-water ratio of 0.70 was selected for XRD, SEM–EDS, and pore size distribution analyses, as shown in Figure 10 and Figure 11. The results indicate that the rapid-setting behavior of geopolymers incorporating VST is still governed by crystallization of the alkali activator. The pore size distribution analysis reveals that, at a VST content of 30%, the pore size distribution becomes more concentrated, and the average pore diameter decreases from 40.46 nm to 12.15 nm. This is likely due to the micro-filling effect of the tailings [48,49], which reduces pore connectivity and the critical pore diameter, thereby improving the pore structure of the geopolymer. Overall, in the vanadium-extraction shale tailings system, the crystallization-induced hardening mechanism of the alkali activator remains applicable, and by appropriately adjusting the experimental parameters, the Si/Al ratio and porosity of geopolymers in the pure metakaolin system can be optimized, leading to the further enhancement of geopolymer performance.

## 4. Conclusions

This study successfully prepared a crystallization-dominated rapid-setting geopolymer by regulating the preparation temperature and the alkali activator modulus. The main conclusions are summarized as follows:

(1) At a preparation temperature of 0–10 °C and an activator modulus of 1.0, the geopolymer achieves a more favorable balance between curing rate and macroscopic strength. The final setting time is 20 min, and the 7-day compressive strength reaches 28.95 MPa, representing a 91.63% reduction in setting time compared with the system prepared at ambient temperature.

(2) The rapid hardening mechanism of the rapid-setting geopolymer differs fundamentally from that of conventional systems. Under low-temperature and low-modulus conditions, the system initially forms a skeletal framework through rapid physical crystallization of hydrated sodium silicate, rapidly converting free water into crystallization water. As a result, the paste loses its fluidity and undergoes rapid setting. The reaction pathway of the rapid-setting geopolymer in this study can be described as “physical crystallization-induced hardening → dissolution and gelation reactions,” which is distinctly different from the traditional “dissolution → gelation → hardening” pathway of conventional geopolymers.

(3) By regulating the incorporation level of vanadium-bearing shale tailings and the alkali-to-water ratio, both rapid setting and enhanced compressive strength can be achieved. With a shale tailings content of 30%, an alkali-to-solid ratio of 0.4, and an alkali-to-water ratio of 0.7, the geopolymer exhibits a setting time of 16 min and an increased compressive strength of 38.32 MPa.

This study provides a new perspective for rapid-setting technologies in the field of solid waste valorization and demonstrates promising application potential in low-temperature scenarios such as temporary engineering support, rapid leakage sealing, and 3D printing. In future work, the long-term durability and functionalization of rapid-setting geopolymers still require further investigation.

## Figures and Tables

**Table 1:** Chemical composition of MK and VST (wt.%).

Component	SiO_2_	Al_2_O_3_	Fe_2_O_3_	CaO	MgO	K_2_O	Na_2_O	TiO_2_	P_2_O_5_	S	Cr_2_O_3_
MK	52.15	42.01	1.25	0.099	0.30	1.51	0.072	0.31	0.143	-	-
VST	66.63	2.54	2.38	6.26	0.31	1.21	0.44	-	-	2.49	0.14

**Table 2:** Composition ratios and preparation temperatures for metakaolin-based geopolymer.

Mix ID	MK (g)	Silicate Modulus (SiO_2_/Na_2_O)	Na_2_O·3SiO_2_ (g)	NaOH (g)	Preparation Temperature (°C)
L-GM0.8	222	0.8	64.53	58.67	0–10
L-GM1.0	222	1.0	80.67	53.33	0–10
L-GM1.2	222	1.2	96.80	48.00	0–10
L-GM1.5	222	1.5	121.00	40.00	0–10
L-GM2.0	222	2.0	161.33	26.67	0–10
N-GM0.8	222	0.8	64.53	58.67	20–30
N-GM1.0	222	1.0	80.67	53.33	20–30
N-GM1.2	222	1.2	96.80	48.00	20–30
N-GM1.5	222	1.5	121.00	40.00	20–30
N-GM2.0	222	1.5	161.33	26.67	20–30

**Table 3:** The surface area and pore volume of L-GM0.8, L-GM1.0, and N-GM1.0.

Samples	Surface Area (m^2^/g)	Pore Volume (cm^3^/g)
L-GM0.8	2.48	0.012
L-GM1.0	3.38	0.024
N-GM1.0	18.29	0.162

**Table 4:** T_2_ distributions of L-GM1.0 and N-GM1.0.

Time	L-GM1.0					N-GM1.0	
	Total Peak Area (S_0_)	T_21_		T_22_		T_22_	
		Value (ms)	Peak Area	Value (ms)	Peak Area	Value (ms)	Peak Area (S_0_)
0 min	1839.89	0.13	180.19	9.01	1659.70	6.52	2679.12
10 min	1644.47	0.13	228.95	7.07	1415.52	5.11	2737.29
30 min	1630.39	0.13	196.08	5.54	1434.31	4.01	2769.77
1 h	1649.67	0.11	161.08	3.70	1488.59	3.14	2805.27
5 h	2067.29			2.10	2067.29	0.57	3363.43
10 h	2443.46			1.40	2443.46	0.30	3193.03
24 h	2767.96			1.40	2767.96	0.28	2954.14

## Data Availability

The original contributions presented in this study are included in the article. Further inquiries can be directed to the corresponding author.
